# Continuous Spectrum of Morphologies and Phase Behavior across the Contact Zone from Poly(l-lactide) to Poly(d-lactide): Stereocomplex, Homocrystal, and Between

**DOI:** 10.1021/acs.macromol.3c01815

**Published:** 2023-10-27

**Authors:** Jiaming Cui, Shu-Gui Yang, Ruibin Zhang, Yu Cao, Yubo Wang, Xiangbing Zeng, Feng Liu, Goran Ungar

**Affiliations:** † Shaanxi International Research Center for Soft Matter, State Key Laboratory for Mechanical Behavior of Materials, Xi’an Jiaotong University, Xi’an 710049, P. R. China; ‡ Department of Materials Science and Engineering, Sheffield University, Sheffield S1 3JD, U.K.

## Abstract

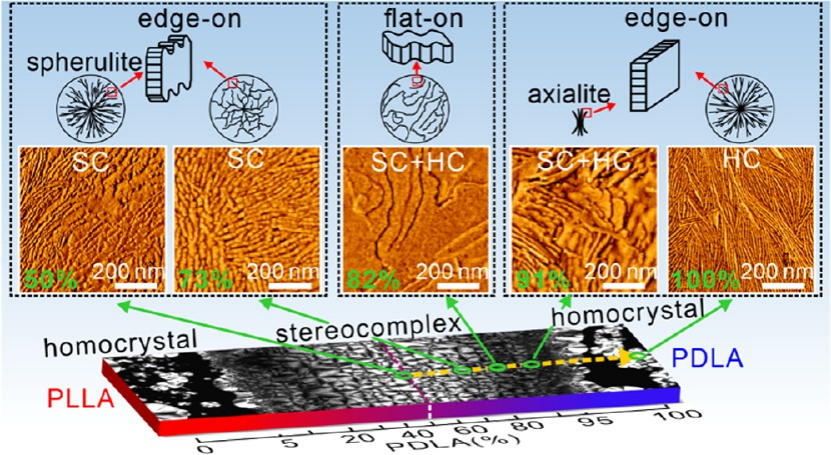

The enantiomeric ratio is a key factor affecting the crystallization behavior and morphology of poly-l-lactide/poly-d-lactide (PLLA/PDLA) blends. Despite a number of studies on crystallization of nonequimolar PLLA/PDLA blends, a full picture of the effect of the L/D ratio is still lacking. Here, we put the two enantiomers in contact and allow interdiffusion above the melting point of the stereocomplex crystal (SC) to prepare samples with a continuously changing L/D ratio from enantiopure PLLA (ratio 0/100) to enantiopure PDLA (100/0). Using polarized optical microscopy, atomic force microscopy, and microbeam X-ray diffraction, the continuous spectrum of morphologies and phase behaviors across the contact zone is investigated. The blend morphology shows clear evidence of “poisoning by purity” of SC crystallization at all blend compositions. The low birefringence of the 50/50 SC is found to be due to the meandering of broken edge-on lamellae. Its further decrease to near zero as L/D deviates further away from 50/50 is explained by transition from radial edge-on lamellae to fully random meandering lamellae, then to mixed flat-on lamellae, and finally to submicron-sized axialites. In comparison with the smooth and straight homocrystal (HC) lamellae of pure enantiomers, the lamellae in the blends often have serrated edges caused by pinning by rejected excess enantiomer acting as an impurity during lamellar growth. A feature of the binary phase diagram is pure enantiomers acting as an impurity to the SC and counter-enantiomer acting as an impurity to homocrystallization of the enantiomers. Crystallization was found to be most suppressed at 99% enantiomeric purity, where the amount of the counter-enantiomer is insufficient for creation of SC nuclei and HC growth is inhibited by the small amount of the enantio-impurity. These and other intriguing results are less likely to be noticed without the continuous composition gradient of the contact sample.

## 1 Introduction

Blending of poly(l-lactic acid) (PLLA) and poly(d-lactic acid) (PDLA) results in the formation of the so-called “stereocomplex” crystal form (SC) in which the PLLA and PDLA chains coexist.^1−3^ SC attracts much attention because it has a significantly higher melting temperature and superior mechanical properties compared to the “homocrystal” (HC) formed by pure PLLA or PDLA enantiomers.^4−9^ Obtaining high-performance PLA products in the SC form is thus of great technological interest. However, achieving this at technologically viable processing speeds still presents many challenges.

Many factors affecting the formation of SC have been investigated over the past three decades, such as molecular weight,^5,10−16^ optical purity,^17,18^ thermal history,^19−21^ and enantiomeric ratio in the blend.^10−13,22−27^ In these studies, the main focus was on the blend ratio. Initially, a L/D ratio of 50/50 was studied extensively, yielding SC crystal structure models with PLLA and PDLA chains packed regularly in the unit cell.^1,2^ Shortly afterward, Tsuji et al. found that exclusively SC is formed not only in the strictly racemic 50/50 blend but also in blends with the ratio ranging from 70/30 to 30/70 (*M̅*_w_ ∼ 3 × 10^4^ g/mol),^11^ which aroused controversy. This has been resolved recently by Tashiro et al., who proposed a structural model with *P*3 symmetry that is highly tolerant to deviations in the enantiomeric ratio. This is because a L/up-L/down (or a D/up-D/down) pair of chains is not too different in shape from an enantiomeric pair D/up-L/up (or D/down-L/down).^3^

Although a considerable amount of research has been done on crystallization of nonequimolar PLLA/PDLA blends, these studies were performed in isolation on selected discrete L/D ratios, by different researchers and on polymers of different molecular weights. This leaves a full systematic picture of composition dependence of crystallization, morphology, crystallinity, and melting behavior of the blends still lacking. Only partial answers have been given to questions such as what is the critical L/D ratio for exclusively SC crystallization,^3^ how does lamellar arrangement change with the L/D ratio,^24^ and what is the physical reason for the weak birefringence of SC spherulites, particularly in nonequimolar blends. In order to give more definite answers, we have prepared samples with a continuously changing L/D ratio from enantiopure PLLA (ratio 0/100) to enantiopure PDLA (100/0) by well-defined contact experiments. The advantage of the present contact method is that all compositions are tested simultaneously on a preparation with constant thickness with all compositions having exactly the same thermal history, molecular weight distribution, etc. Neat PLLA and PDLA films were melted and joined at 250 °C to form a step function in L/D concentration, followed by a fixed-period annealing of the melt at 230 °C allowing PLLA and PDLA molecules to interdiffuse, creating a nearly linear L/D concentration profile of convenient width. The shape of that profile could be determined by measuring the brightness across the contact region under slightly decrossed polarizers. The continuous spectrum of morphologies and phase behaviors across the contact zone prepared by different thermal treatments was studied by using polarized optical microscopy (POM), microbeam wide-angle X-ray scattering (μ-WAXS), and atomic force microscopy (AFM). Complementary experiments on a series of discrete blends covering the entire composition range were done by using differential scanning calorimetry (DSC) and fluorescence microscopy. A consistent scheme is proposed encompassing the wide range of observed phenomena such as highly disturbed serrated crystalline lamellae due to poisoned crystallization, radial vs tangential lamellae, spherulites vs axialites, edge-on vs flat-on lamellae, and one crystal form nucleating another.

## 2 Preparation of PLLA/PDLA Contact Samples with the Composition Gradient

**Figure 1:**
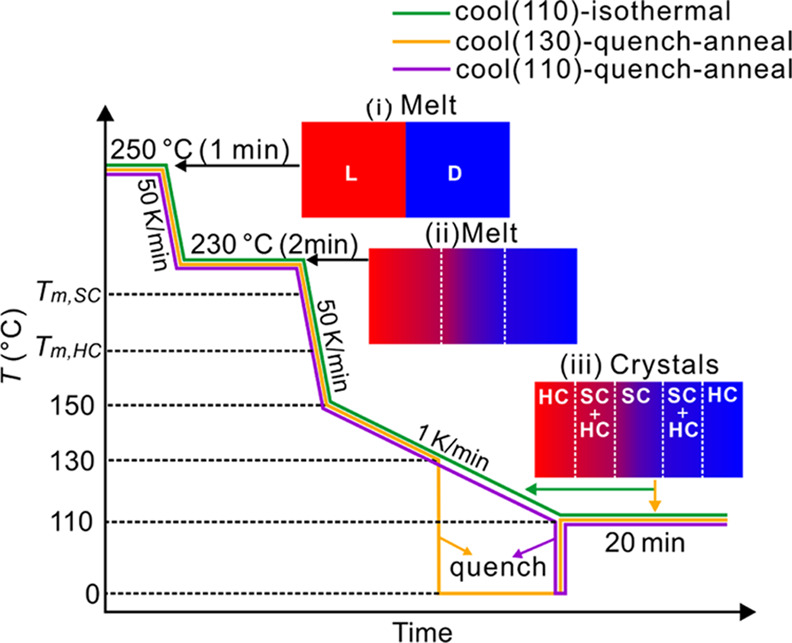
Three temperature–time protocols for the preparation of crystalline PLLA/PDLA contact samples with continuously varying L/D ratio. Sample designation and the color codes are shown at the top right. Insets (i, ii) depict the interdiffusion between PLLA and PDLA, while (iii) shows the distribution of crystal forms in the contact sample.

The contact samples were designed to provide a continuously increasing fraction of one enantiomer in another from 0 to 100%. The detailed procedure is outlined in Figure 1. First, the PLLA and PDLA films were brought into contact at 250 °C on a microscope slide covered with a coverslip and held at this temperature for 1 min. Subsequently, the sample was rapidly cooled to 230 °C, i.e., just above the melting point of SC, and kept at this temperature for 2 min. This established a composition gradient between the two melts. Then, the sample was cooled quickly (50 K/min) to 150 °C, followed by slow cooling (1 K/min) to 130 °C. Once the temperature was below 150 °C, SC spherulites formed in the contact region. HC spherulites in both contact and pure regions mainly formed below 130 °C. Three routes were followed, shown with green, orange, and purple lines: (a) The “cool (110)–isothermal” route (green, to form large HC spherulites): sample was cooled to 110 °C at 1 K/min and then held at that temperature for 20 min. (b) The “cool (130)–quench–anneal” route (orange, to avoid the formation of large HC spherulites): upon reaching 130 °C, the sample was quenched in ice water and then annealed at 110 °C for 20 min. (c) The “cool(110)–quench–anneal” route (purple): this ensures complete crystallization even in the border area between the blend and pure enantiomer where the melting point is the lowest.

**Figure 2:**
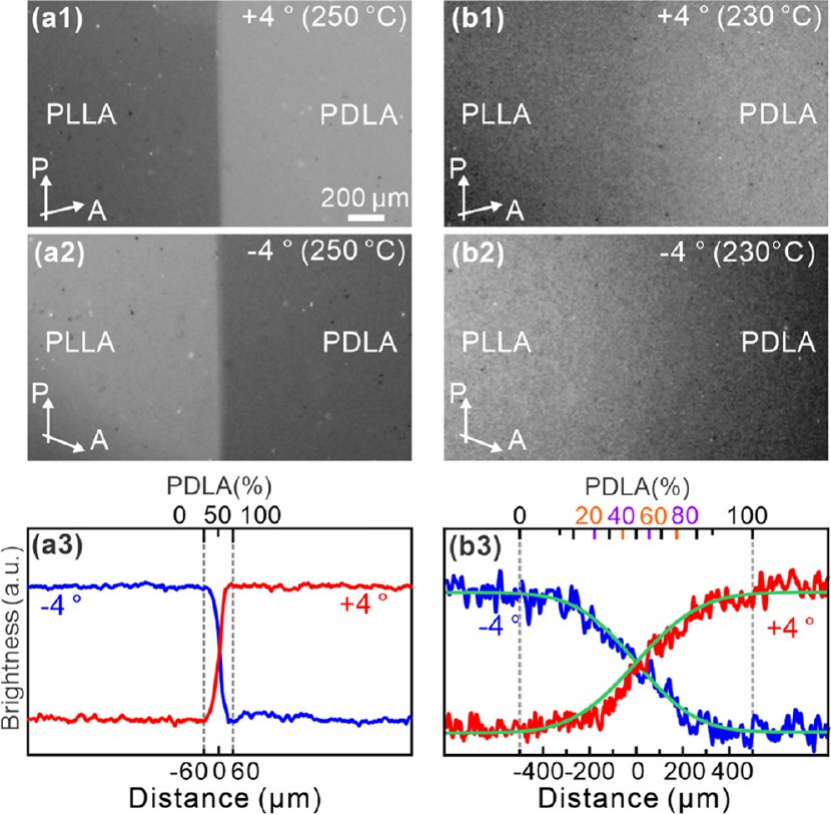
Polarized micrographs of a contact sample recorded after PLLA and PDLA had been in contact (a1 and a2) at 250 °C for 5 s and (b1 and b2) after annealing at 230 °C for 2 min. The analyzer was decrossed (a1, b1) by +4° and (a2, b2) by −4°. (a3, b3) Plots of brightness, averaged along the entire height of image, vs distance from the contact line. The green line is a fit to the error function (*erf*), the solution of the Fick diffusion equation for a flat interface. The alternative abscissa scale is % of PDLA.

The optical rotation angle φ of molten PLLA and PDLA was determined by measuring brightness as a function of deviation of the analyzer angle from 90° to the polarizer (referred to as decrossed). The analyzer was rotated from −12° to +12° away from the crossed orientation in 1° steps, and images were taken in rapid succession. The measured φ was approximately +1° for PLLA and −1° for PDLA. The difference in brightness between PLLA and PDLA is greatest at ±4° (see Figure S1a,b of the Supporting Information, SI). Thus, in order to determine the composition gradient between PLLA and PDLA melts, optical images of the contacted PLLA/PDLA sample under decrossed polarizers (±4°) were captured at 250 and 230 °C. A clear sharp boundary between PLLA and PDLA melts can be seen at 250 °C immediately upon the establishment of the contact (Figure 2a1–a3), whereas after 2 min at 230 °C, the boundary between the two enantiomers is blurred (Figure 2b1–2b3). The composition profile across the contact region was determined by fitting the brightness plots with an error function (*erf*), the solution of the Fick diffusion equation for a flat interface (Figure 2a3,b3 (note the top abscissa scale)).

## 3 Results

### 3.1 Crossed-POM Observations

**Figure 3:**
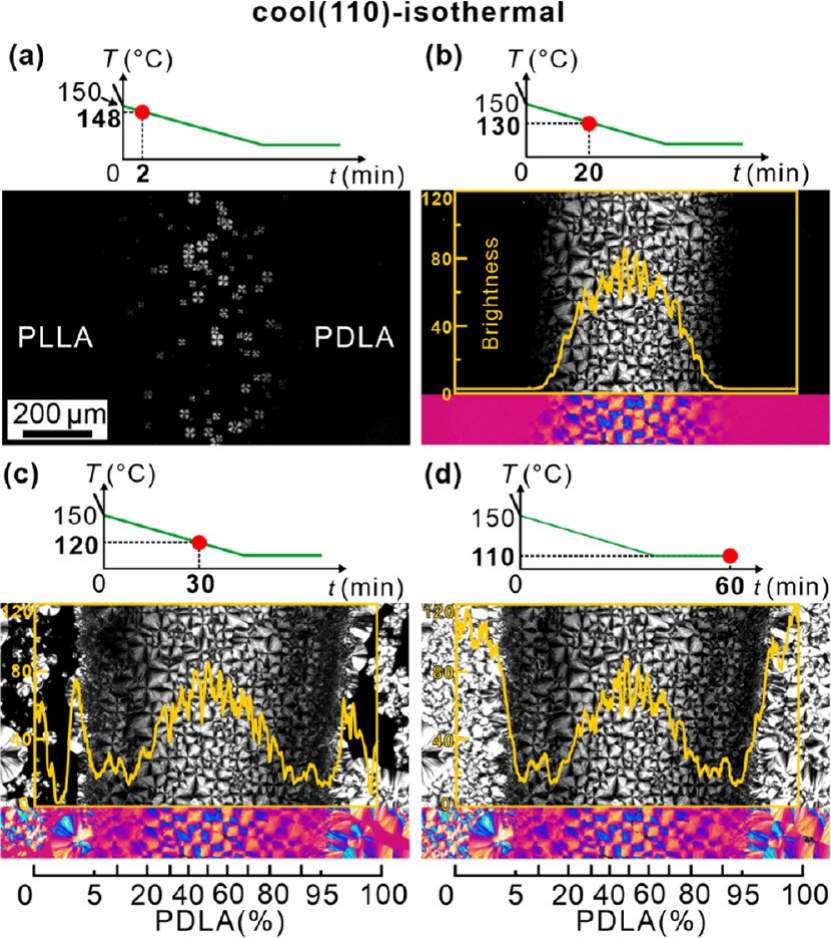
POM images of “cool (110)–isothermal” sample recorded at (a) 148 °C, (b) 130 °C, and (c) 120 °C while cooling from 150 °C at 1 K/min. (d) Recorded after holding the sample for 20 min at 110 °C. In this figure and Figure 5, the bottom part of (b–d) was replaced by matching area micrographs taken with a λ-plate in order to determine the orientation of the slow axis and thus the “sign” of the spherulites, i.e., “negative” (slow axis tangential) or “positive” (slow axis radial). In all areas, the spherulites are seen to be “negative”. Similarly, in both this and Figure 5, the yellow curves in (b–d) are line profiles of brightness integrated along the height of the yellow rectangle.

Figure 3 shows the POM images of the “cool (110)–isothermal” sample recorded at different temperatures. Once the temperature is below 150 °C, birefringent spherulites with the Maltese cross start to form in the contact region, while the regions of pure enantiomers remain molten. The spherulites that did form at 150 °C were SC, which will be confirmed by melting experiments in Section 3.2 and μ-WAXS in Section 3.3. Figure 3a shows SC spherulites at 148 °C, the diameter of which ranges from 20 to 40 μm. As the temperature decreased to 130 °C, these spherulites kept growing and impinging, finally forming a birefringent band in the contact region (Figure 3b). Micrographs with a λ-plate show that all SC spherulites were optically negative, regardless of the L/D ratio. The SC spherulites were brightest at L/D 50/50, the birefringence deceasing gradually with L/D deviating further away from 50/50. This phenomenon has already been reported by Tsuji et al.^12,22^ and Woo et al.,^28^ who studied discrete samples with different L/D. To the best of our knowledge, the cause of the decreasing birefringence has not yet been reported; it will be discussed further below.

With a further decrease in temperature to 120 °C (Figure 3c), HC spherulites appear in both enantiomeric regions, with birefringence stronger than that of SC of any composition. However, a narrow region with L/D between 99/1 and 100/0 (and between 1/99 and 0/100) still remains molten even at 120 °C (Figure 3c). At this temperature, the melt is highly supercooled, and pure enantiomers normally crystallize in the HC α-form very readily.

**Figure 4:**
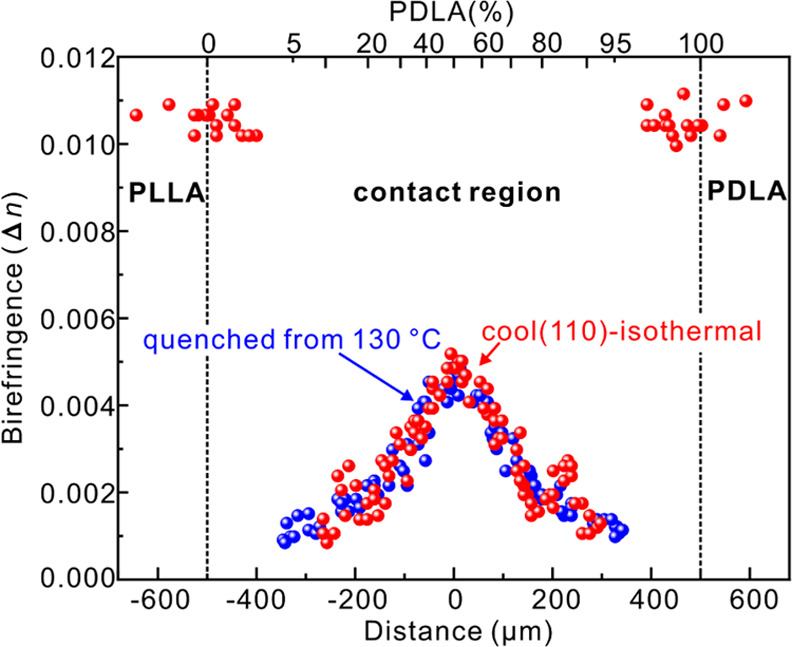
Birefringence at 25 °C of the “cool (110)–isothermal” sample and sample quenched from 130 °C as a function of distance from the contact line (top abscissa scale shows the L/D ratio).

As the temperature decreases further to 110 °C, HC spherulites start forming in the 99/1–100/0 region as well. After maintaining at 110 °C for 20 min, the whole field is filled with spherulites, even though the birefringence of some areas is very weak indeed; see Figure 3d. The brightness curve clearly shows that HC spherulites are brighter than SC ones even at their brightest at L/D 50/50. In order to obtain a more quantitative measure of spherulite birefringence, Δ*n* was measured across the composition spectrum using a Berek compensator, with the results plotted in Figure 4. Δ*n* of HC α-form spherulites is seen to be twice that of SC at L/D 50/50.

**Figure 5:**
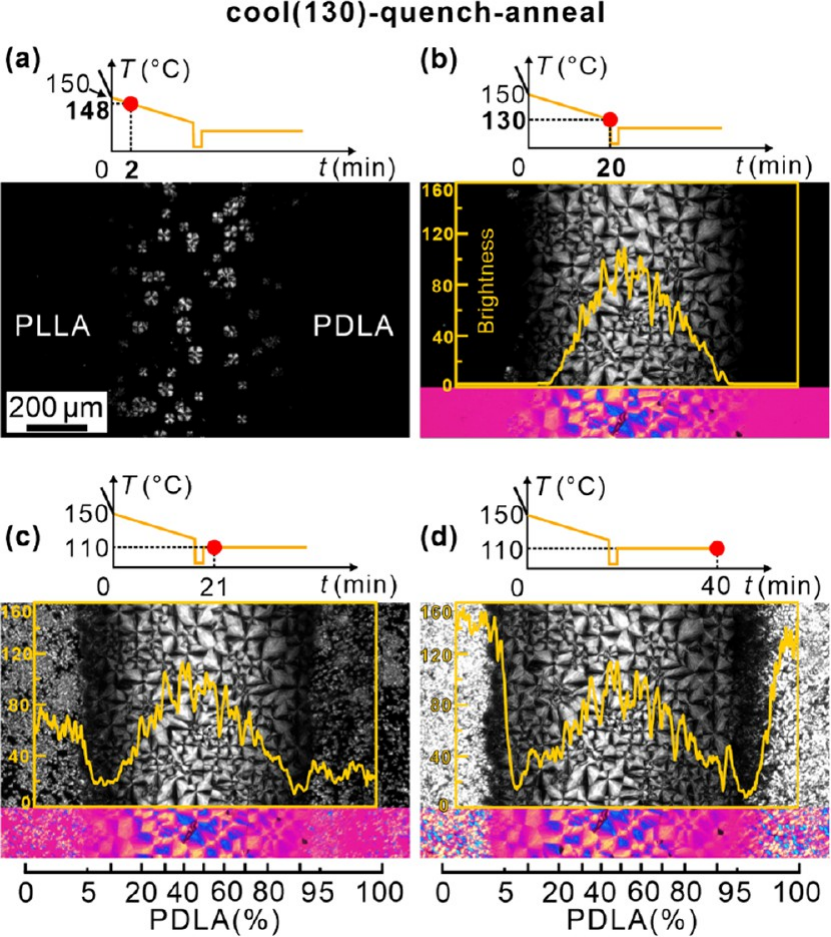
POM images of the “cool (130)–quench–anneal” sample recorded during cooling from 150 °C at 1 K/min: (a) 148 °C and (b) 130 °C. (c, d) Recorded after quenching from 130 °C to room temperature and subsequent annealing at 110 °C for 1 and 20 min, respectively.

Figure 5 shows polarized micrographs of the “cool (130)–quench–anneal” sample. As expected, Figure 5a,b is very similar to Figure 3a,b, recorded after an identical thermal cycle. However, unlike in Figure 3c, where the slow cooling had continued to below the crystallization temperature of HC, Figure 5c shows the situation after the slow cooling was interrupted by quenching and subsequent annealing for 1 min at 110 °C, well above the *T*_g_. The multitude of nuclei in areas of pure enantiomers produce many large axialites, which fill the space fully on annealing for a further 19 min (Figure 5d).

### 3.2 Change in Brightness on Cooling and Reheating

**Figure 6:**
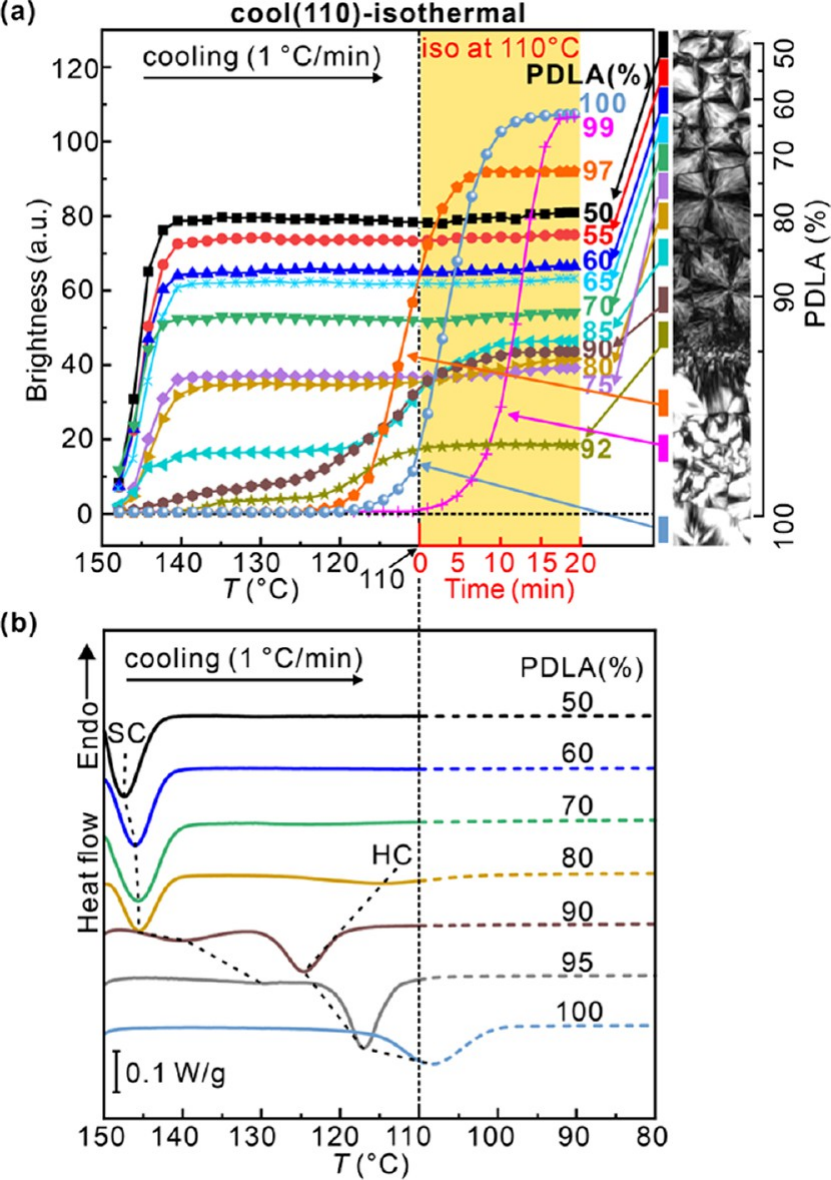
(a) Change in brightness of regions with different L/D ratios during the “cool (110)–isothermal” process, i.e., during cooling from 150 to 110 °C at 1 K/min followed by isothermal annealing at 110 °C for 20 min (area shaded yellow). A strip of the contact sample polarized micrograph is shown on the right with bars color-coding the different regions. The two step-ups in brightness correspond to SC and HC crystallization. Note the anomalous behavior in the 90–100% range. (b) DSC thermograms on continuous cooling to 80 °C for discrete samples with compositions in the same range as in (a), using the same color codes.

Brightness of different L/D regions of POM images (50–100% PDLA region) was measured during cooling and plotted vs temperature in the left-hand (white) part of Figure 6a. The values were averaged over the full height (vertical) of the contact image and the width (horizontal) of the particular L/D section. The right-hand (yellow) part of the diagram shows the continued development during isothermal annealing at 110 °C. Within the PDLA content range of 50–75%, brightness increases steeply at first on cooling at 1 K/min, soon reaching saturation at ∼140 °C as SC crystallization completes. The corresponding DSC cooling curves at 50, 60, and 70% (Figure 6b) contain the expected SC crystallization exotherm coincident with the step increase in brightness. Both results confirm previous reports^3,11,26^ that only SC forms in the L/D range of 70/30–30/70. However, we note that this applies only to slow cooling rates. At higher rates, such as 5 K/min, SC crystallization of the medium-molecular weight racemate (54 kDa) stops in its tracks soon after commencement, around 140 °C, and only continues at 120–110 °C as HC crystallization.^29^ This unusual “hesitant” behavior was recently explained by a “poisoning-by-purity” effect, whereby due to compositional fluctuations, a local excess of one enantiomer is excluded from the crystals and accumulates at the growth front, blocking its further progress. However, at low cooling rates, such as 1 K/min, such pileups are resolved through molecular diffusion. Nevertheless, evidence of such a poisoning effect even at slow cooling is abundant in morphology, as shown further below. Consistent with the above-mentioned model, it was found that such poisoning is much less pronounced in polymers with a lower molecular weight (e.g., 20 kDa),^29^ doubtless because of the increased diffusion rate. Conversely, SC crystallization is even more suppressed in high*-M̅*_w_ PLA racemates.^12,14^

The two-step brightness curves and two-exotherm DSC curves in the 80–95% range are consistent with a decrease in the amount of SC crystallization and a concurrent increase in the level of HC crystallization. One can also notice that the jump in brightness due to HC crystallization in almost all blends happens earlier, i.e., at higher temperature, than in neat PDLA, indicating a nucleation effect of SC on HC crystallization. This has already been noted by several researchers.^13,30−33^ The exception is the 99% blend, which crystallizes the latest of all. Clearly, the benefit of SC nuclei is gone at that composition, yet there is still sufficient PLLA enantiomer to act as a poison for PDLA HC nucleation. From the observed behavior, we can conclude that the presence of a small fraction of the enantio-impurity is beneficial for rapid crystallization of the PLA homopolymer.^30,31^

**Figure 7:**
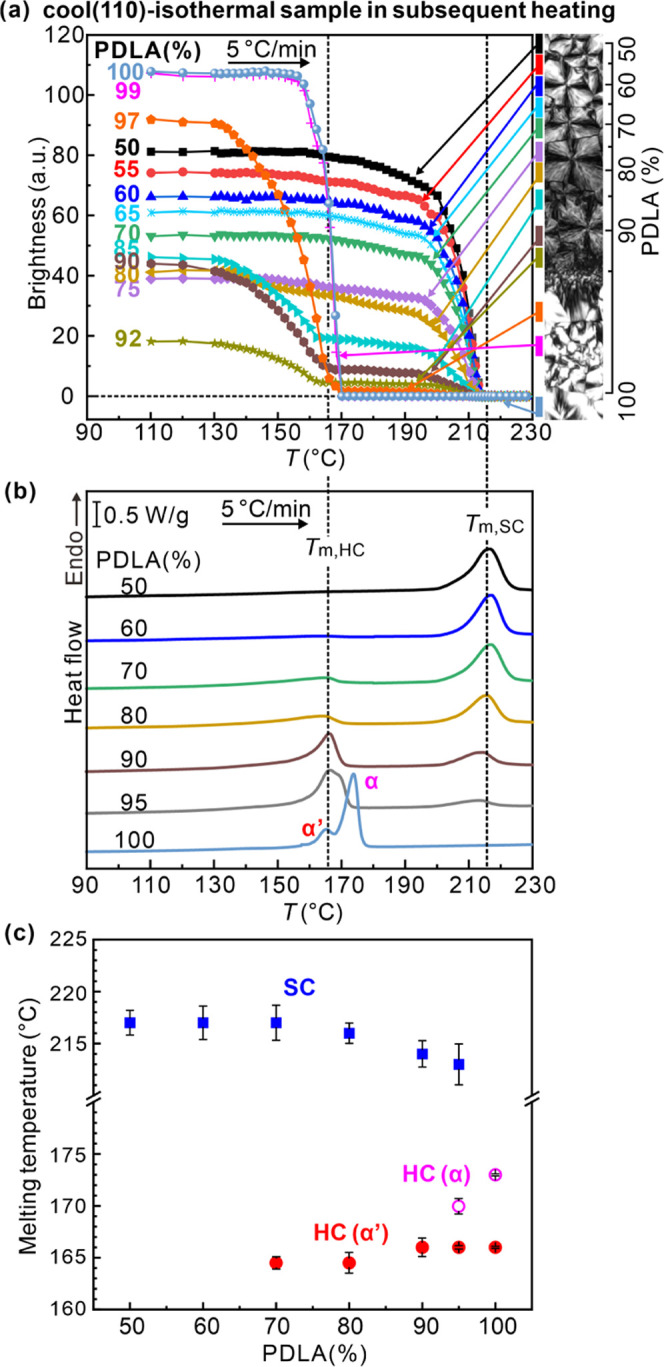
(a) Brightness vs temperature during heating at 5 K/min of the “cool (110)–isothermal” sample. (b) Mirrored DSC heating traces of separately prepared blends; percentages of PDLA are indicated. (c) Melting temperatures of SC and HC from DSC traces in (b).

The melting behavior of different L/D blends was also studied by POM. The “cool (110)–isothermal” sample was heated to the temperature at which last birefringence disappeared while recording POM images. Brightness values at different L/D ratios are plotted vs temperature in Figure 7a. For PDLA fractions between 50 and 75%, no downward step is seen in the temperature range of HC melting, consistent with the crystallization curves in Figure 6. A gradual premelting decrease in brightness, starting at around 160 °C, ends in a sharp loss in the SC melting range around 210 °C. From 80% onward, premelting ending with a sharp drop in brightness at 165 °C is also observed signaling melting of HC.

DSC heating curves of separately prepared PLLA/PDLA blends with discrete L/D ratios are shown in Figure 7b. The HC and SC melting endotherms, with peak temperatures *T*_m,HC_ and *T*_m,SC_, are in agreement with the drops in brightness in Figure 7a, except that a small amount of HC melting is observed already at 70% PDLA. *T*_m,HC_ and *T*_m,SC_ values are plotted against the composition in Figure 7c. The observed decrease of *T*_m,SC_ and increase in *T*_m,HC_ are as expected: deviation from 50% PDLA is seen as an impurity by the SC, while deviation from 100% is seen as impurity by the HC. In fact, it may appear unusual that the decrease in *T*_m,SC_ is as small as seen in Figure 7c, but the high tolerance to compositional imbalance of the SC has been aptly explained by Tashiro et al.^3^

It is notable that the melting endotherm of HC in 100 and 95% PDLA is a doublet, with a higher temperature second peak or shoulder at 170 °C. We attribute the higher temperature peak to the melting of the α-form and the lower temperature peak to that of the α′-form with a less compact unit cell;^34,35^ see the next section.

### 3.3 Dependence of the Crystal Form on the L/D Ratio

**Figure 8:**
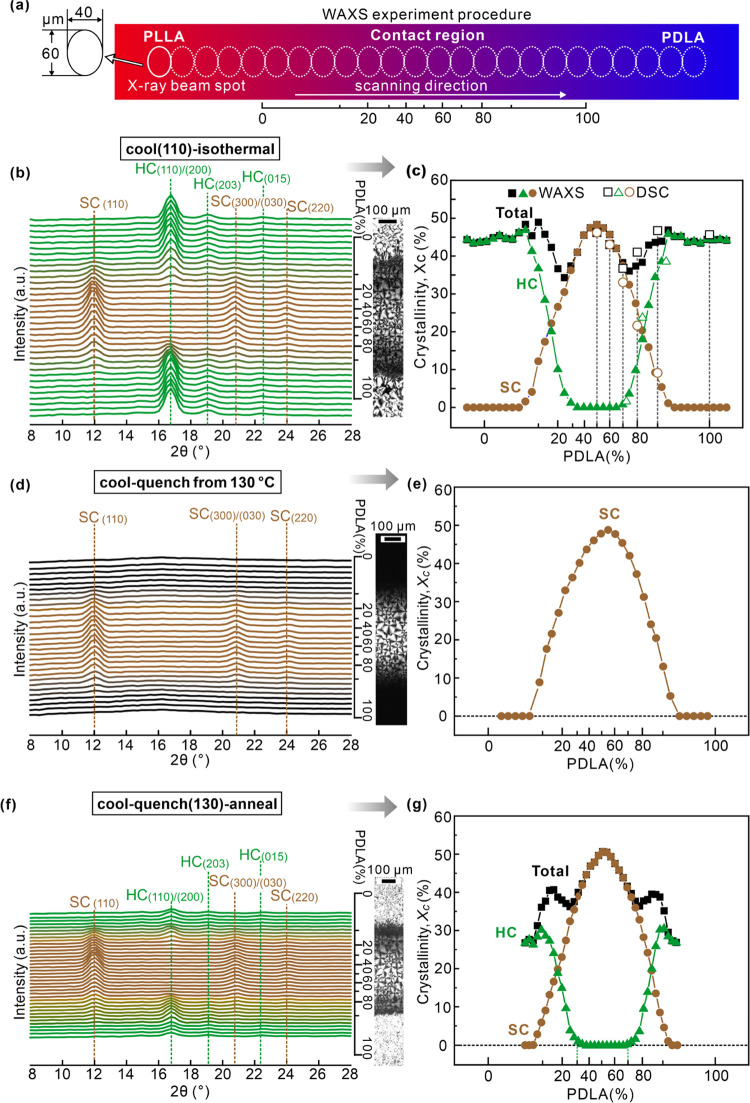
(a) Schematic representation of the geometry of the WAXS scan. The ellipses, scaled to the sample length, represent the 40 × 60 μm^2^ X-ray beam spot at the sample. WAXS diffractograms recorded at successive positions, are plotted in (b) for the “cool (110)–isothermal” sample, (d) for the sample quenched in ice water once a temperature of 130 °C was reached on cooling at 1 K/min from 150 °C and (f) for the “cool (130)–quench–anneal” sample. (c,e,g) Plots of crystallinity of SC and HC and their sum obtained from (b, d, f). The crystallinity obtained from DSC using the same temperature program as for the “cool (110)–isothermal” sample was also provided for comparison in (c).

The crystal form as a function of the L/D ratio in the contact samples was investigated by μ-WAXS. As shown in Figure 8a, the scanning direction is perpendicular to the centerline of the contact/diffusion region. The long axis of the elliptical beam cross-section was perpendicular to the scanning direction. Figures 8b,c,f shows the WAXS diffractograms recorded at different positions, corresponding to the POM strips on the right. Three contact samples were scanned, having different thermal histories: “cool (110)–isothermal” (Figure 8b,c), “cool–quench from 130 °C” (Figure 8d,e), and “cool (130)–quench–anneal” (Figure 8f,g). As the L/D ratio deviates from 50/50, characteristic reflections of SC, i.e., SC_(110)_, SC_(300)/(030)_, and SC_(220)_, gradually fade away. At the same time, in “cool (110)–isothermal” and “cool(130)–quench–anneal” samples, HC_(110)/(200)_, HC_(203)_, and HC_(015)_ diffraction peaks intensify.

Figure 8c shows the *X*_SC_, *X*_HC_, and total crystallinity (*X*_total_ = *X*_SC_ + *X*_HC_) of the “cool (110)–isothermal” sample as a function of the L/D ratio. *X*_SC_ and *X*_HC_ are defined as fractions of the two crystal forms in the total material. *X*_SC_ is seen to decrease monotonously as the L/D ratio departs from 50/50. *X*_HC_ emerges at about 30/70 and 70/30, agreeing with DSC results (Figure 7b). With the L/D ratio deviating further from 50/50 (L/D < 30/70 and >70/30), HC composed of α and α′ (see Figure S2 of the SI) emerges and gradually increases up to a plateau level (at L/D = 8/92), consistent with the double melting endotherms around 170 °C in Figure 7c. Interestingly, *X*_total_ decreases at first and then increases again as a pure enantiomer is approached, passing through a minimum around L/D 30/70 and 70/30. This is in agreement with DSC crystallinity, which at 30/70 is lower than at 40/60 and 20/80. *X*_SC,DSC_, *X*_HC,DSC_, and *X*_total,DSC_ are marked in Figure 8c with hollow symbols.

1D WAXS profiles of the “cool–quench from 130 °C” sample are shown in Figure 8d. The figure shows that the only crystals formed were SC, which was in the contact region. After annealing the quenched sample at 110 °C for 20 min, HC had formed in the regions with ∼L/D < 30/70 and >70/30, as shown in Figure 8f. It again shows the minimum in *X*_total_ around 30/70 and 70/30. Besides, HC that formed in neat enantiomers after annealing at 110 °C has a much lower crystallinity than in the “cool (110)–isothermal” sample (Figure 8g).

### 3.4 Fluorescence Microscopy

Adding a fluorescent dye to polymers and monitoring its distribution during or after crystallization using fluorescence microscopy is a versatile technique for imaging the morphology of semicrystalline polymers, both in two-dimensional (2D)^36^ and in three-dimensional (3D).^37−39^ The imaging is based on rejection of the dye from crystal lamellae and from the spherulite as a whole, thus providing the necessary contrast. 2D images can be obtained by using a conventional reflection optical microscope equipped with a suitable exciting light source and dichroic mirror and filters. 3D imaging, or optical tomography, can be performed by using laser scanning confocal microscopy.

**Figure 9:**
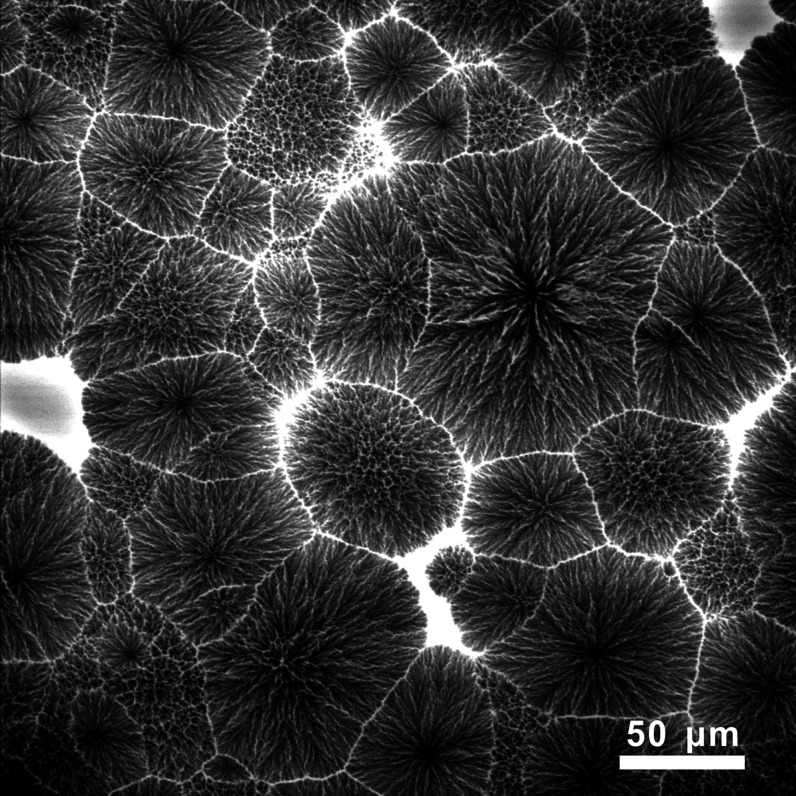
Two-photon laser scanning confocal fluorescence micrograph. A thin *xy* slice from the interior of a 20/80 PLLA/PDLA blend (*M̅*_w_ of PLLA is 9 kDa and PDLA is 7 kDa) containing 0.05 wt % Nile Red cooled from 260 to 135 °C at 20 K/min and left to crystallize isothermally for 10 min. Dendritic SC spherulites are dark. Bright areas are rich in the dye, which had concentrated in remaining molten areas of nearly pure excess PDLA rejected from the growing SC lamellae. These bright areas are the “interfibrillar” regions within the spherulites and interspherulite boundaries.

Figure 9 shows a *xy* slice at a suitable depth *z* of a 20/80 L/D sample containing 0.05 wt % Nile Red. The sample was isothermally crystallized at 135 °C and then quenched in ice–water. The dendritic-like spherulites are SC, while the bright regions are amorphous glass, where the excess PDLA had aggregated and remained molten at 135 °C, containing the dissolved dye. Note the meandering lamellae reflecting the tortuous trajectory of SC crystal growth amid excess nearly pure PDLA acting as impurity to SC. The morphology supports the “poisoning-by-purity” model.^29^

**Figure 10:**
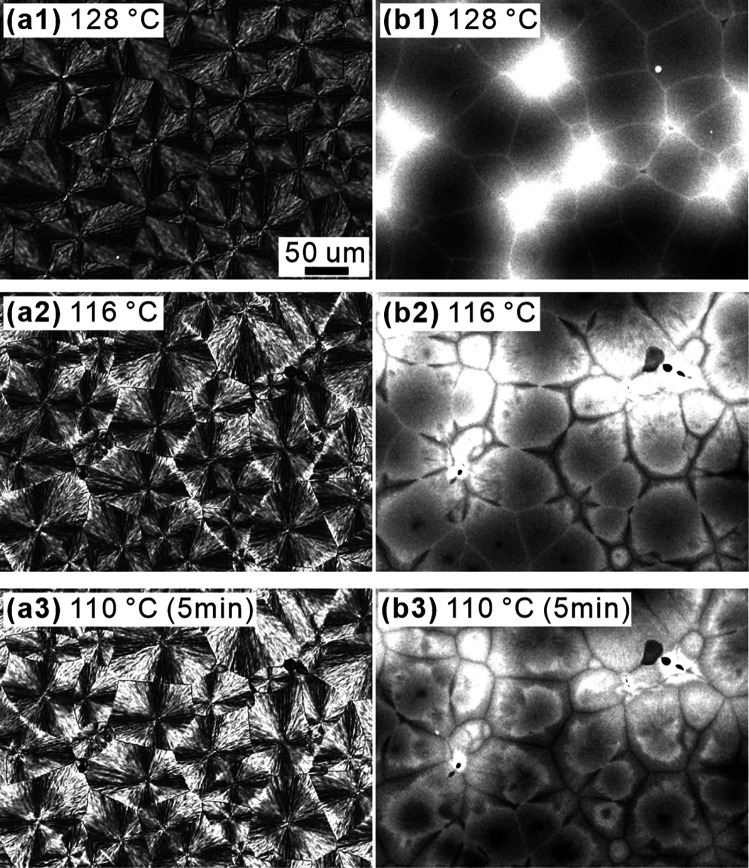
(a1–a3) Polarized optical micrographs of 20/80 blends. *M̅*_w_ values of PLLA and PDLA are both 54 kDa. The dye-labeled PLLA/PDLA blend recorded during cooling at 1 K/min. (a1) 128 °C, (a2) 116 °C, and (a3) held at 110 °C for 5 min. (b1–b3) Corresponding fluorescence micrographs of the same area taken immediately after the POM.

The same L/D 20/80 dye-labeled blend was also investigated by POM and 2D fluorescence microscopy. Figure 10 shows a series of micrographs recorded at different temperatures during slow cooling. The left are POM images, and the right are the corresponding fluorescence images taken from the same area immediately after the images on the left. In Figure 10a1,b1, only SC had crystallized, POM showing the typically low-birefringent SC spherulites. In b1, thin bright lines can be seen between spherulites, representing the remaining melt containing nearly pure PDLA and the concentrated dye. However, on reaching 116 °C, those bright spherulite boundaries turned dark (Figure 10b2) as pure PDLA had now crystallized as HC and expelled the dye back into the loosely packed SC spherulite. At the same time, thin bright spherulite boundary lines started to appear in the POM image (Figure 10a2), representing the more birefringent HC forming at those boundaries. This effect is more pronounced after 5 min at 110 °C (Figure 10a3), as more of the remaining PDLA enantiomer crystallized in those areas. The spherulites themselves also became much brighter, as PDLA crystallizes also between the lamellar stacks of the original SC spherulites. POM observations, similar to those in Figure 10a1–a3, have also been reported by Tsuji and Ikada^12^ The dark spherulite boundaries in the fluorescence image (Figure 10b3) have widened as continued crystallization of PDLA kept expelling more dye into the peripheral regions of SC spherulites, giving them a diffuse bright rim.

### 3.5 Lamellar Morphology by AFM

From the above-mentioned images and from direct measurement, it is clear that the birefringence of SC is significantly lower than that of HC and that it decreases further on moving away from the 50/50 composition to virtually zero close to the SC–HC boundary. To understand this and other observations described above, AFM was used to observe the morphology at a higher resolution.

**Figure 11:**
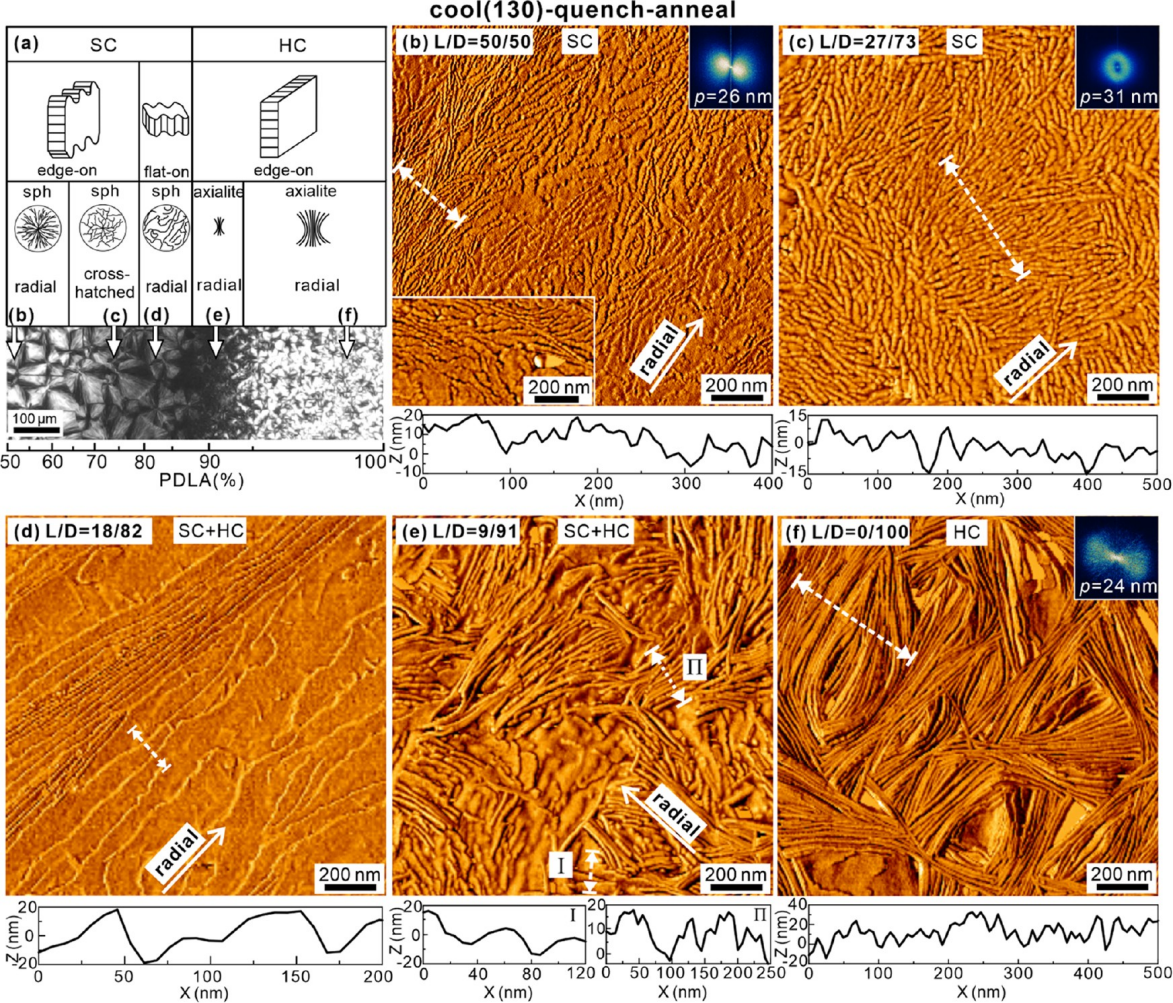
AFM of “cool (130)–quench–anneal” sample. (a) Schematic diagram of the lamellae (top row) and POMs (middle row) of the right-hand side of the sample, corresponding to the different regions on the POM strip (bottom row). Positions (b–e) are marked at which AFM images in panels (b–e) were taken. (b–e) AFM phase images of morphologies recorded at positions with different L/D ratios. (b) 50/50, (c) 27/73, (d) 18/82, (e) 9/91, and (f) pure PDLA. In this and subsequent figures, insets show FT patterns. The lamellar period (*p*) is estimated from FT. Height scans at the bottom of each image were recorded along the white dashed lines in the corresponding height images (Figure S3 of the SI).

AFM phase images of spherulites recorded at different positions along the composition gradient of the “cool (130)–quench–anneal” sample are shown in Figure 11. The scanning positions are indicated by arrows in the POM image of the 50–100% PDLA side of the contact sample in Figure 11a. The corresponding height images are shown in Figure S3 of the SI.

Figure 11b is the AFM picture of a part of an SC spherulite with L/D ∼ 50/50. The lamellae are roughly radial, generally oriented southwest-northeast, as is also obvious from the Fourier transform (FT) in the inset at the top right. The lamellae are mainly edge-on. The remarkable feature is their meandering trajectory, with many breaks and short runs. In many places, one could also see periodic bulges on the protruding lamellar edge. In a few areas where the lamellae are tilted or flat-on, as in the inset at the bottom of Figure 11b, these edges are seen to be serrated, sawtooth-like. These are the clearest indications of the pinning during lamellar growth caused by poisoning by excess enantiomer. Compare this with the edges of HC lamellae in the pure enantiomer in Figure 11f that are smooth and straight.

Figure 11c was recorded at a position with ∼73% PDLA. Interestingly, here, the lamellar orientation is mixed, both radial and closer to tangential. The coexistence of radial and tangential lamellae has also been reported by Prud’homme and Wang.^24^ In their case, they even observed birefringence reversal, with the slow axis turning from tangential to radial in the spherulites, as tangential lamellae dominated. In our case, this does not happen, but the birefringence in these areas drops to near zero. The loss of birefringence is also consistent with the very small, subwavelength size of oriented domains seen in Figure 11c. As shown by the FT inset, the lamellar orientation even in such a small image (only ∼1 μm) is already practically random. As indicated by lamellar randomization, away from the 50/50 composition, poisoning of SC growth by excess enantiomer is enhanced even further.

Figure 11d shows an area with L/D ∼ 18/82. Here, both flat-on and edge-on lamellae are seen. At this L/D ratio, after crystallization of SC, HC starts to crystallize from ∼130 °C onward on cooling (Figure 6). It is hard to say if the lamellae in the picture are SC or HC. Based on the fact that they are rather large and their edges are less rugged than those in Figure 11b,c, they are more likely to represent HC of PDLA. At this composition, smooth lamellar edges are not expected for either SC or HC because not only is excess PDLA impurity for SC but also, equally, the presence of PLLA is impurity for PDLA HC. Furthermore, flat-on lamellae are a sign of low nucleation rate relative to growth rate, as lamellae far away from the nucleus are likely to lie parallel to the surface of the thin film. At the temperature at which these crystals had grown, supercooling for HC is very much smaller than for SC, and hence, HC nucleation is expected to be much scarcer than nucleation of the highly supercooled SC. Figure 11d may also be compared to Figures 9 and 10, all representing a similar L/D composition. The increased brightness on annealing the quenched samples in Figure 10a obviously comes from the edge-on lamellae of HC.

In the region of 91% PDLA (Figure 11e), axialites on the order of 1 μm are seen. Interspersed between lamellae with smooth edges are those with periodic protrusions, i.e., with serrated edges. We tentatively attribute the former to HC crystals of PDLA and the latter to a few SC lamellae still able to grow in pockets of sufficiently high concentration of PLLA. The absence of serrated lamellae in the region of pure enantiomer PDLA (Figure 11f) is consistent with the above-mentioned morphological assignments.

**Figure 12:**
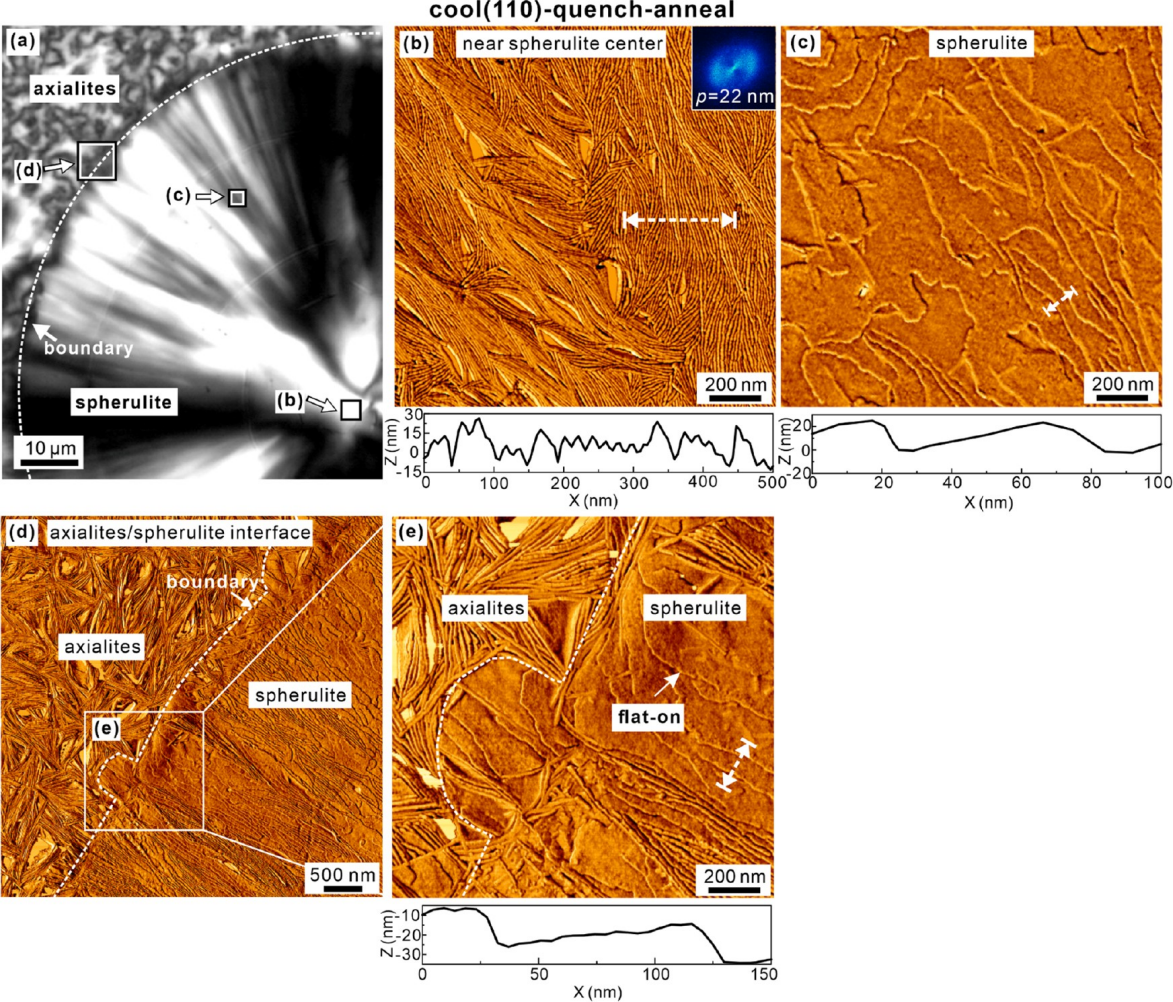
AFM of the “cool (110)–quench–anneal” sample. (a) POM image of a large HC spherulite of 100% PDLA with small axialites at the top left at the border with the PDLA/PLLA blend. (b–e) AFM phase images of square-boxed regions indicated by arrows in (a). (b) Near the center of the spherulite; (c) dark area in the dark slice in POM in the northwest sector; and (d) interface between spherulite and axialites. (e) Enlarged white rectangle in (d). Height scans of each image were recorded along the white dashed lines in the corresponding height images (Figure S4 of the SI).

Unlike the sample that was slowly cooled to 110 °C followed by isothermal annealing at that temperature (Figure 11), the contact sample shown in Figure 12 had been quenched in ice water when the temperature reached 110 °C and then reheated back to 110 °C and continued crystallizing isothermally, the “cool (110)–quench–anneal” sample (Figure 1). This ensured complete crystallization of the sample, even in the border area between pure enantiomers and neighboring areas with a small amount of enantiomer impurity. The POM image in Figure 12a shows a large HC spherulite nucleated still during initial slow cooling and another area (top left) full of small aggregates nucleated on quenching. Positions where AFM images were taken are marked on the POM picture in Figure 12a. The AFM image in Figure 12b was taken in a bright area near the center of the large spherulite. It shows densely packed, smooth, edge-on, mainly radial lamellae. Figure 12c shows clearly why slice (c) within the mainly bright sector of the spherulite is dark; the lamellae, still radial, are seen lying flat-on. Figure 12d,e shows a dark borderline area of the large spherulite. Lack of birefringence on the spherulite side of the border is seen to be due to flat-on lamellae, while outside the border, it is due to the size of the axialites being too small to generate light of sufficient net ellipticity to pass through the analyzer. Interlamellar spacing of edge-on lamellae as well as step height at edges of flat-on lamellae, both determined from height scans, are all close to 30 nm.

## 4 Discussion

Applying the contact method in optical microscopy is an established technique in studies of liquid crystals as a way of finding whether an unknown phase X in compound 1 is the same as a known phase Y in reference compound 2. The absence of a phase boundary is then an indication that X = Y. The contact method is not commonly used in polymers, but the present work illustrates its usefulness in certain situations. While a number of features of PLLA/PDLA blends observed here have been reported previously,^12,24,25^ the contact method increases reliability by isolating the effect of composition from any other factors that may interfere, as all other conditions are kept exactly the same. In addition, the danger of missing certain effects confined to a narrow range of compositions is minimized due to the continuity of the composition spectrum.

### 4.1 Morphological Evidence of Poisoned Crystallization

A most striking feature highlighted by this work is the difference in morphology between the blends and the pure enantiomers, shown by AFM images of different areas of the contact specimens. Highly disordered, winding, and serrated lamellae are seen at all compositions of less than about 90% enantiomeric purity (Figure 11). Such disorder even for the 50/50 racemate, a composition considered ideal for stereocomplex growth, is the clearest demonstration of the “poisoning-by-purity” effect.^29^ As mentioned above, the effect is attributed to local compositional fluctuations in the crystallizing melt, leading to blockage of growth by the accumulated rejected excess enantiomer. The fact that birefringence of crystalline blends at its highest, i.e., at L/D = 50/50, is only half that of the pure enantiomer (Figures 3 and 5), even though the crystallinities are the same (Figure 8c), is clearly caused by the fractal nature of lamellar texture in blends, on a scale comparable to or smaller than the wavelength of light.

The fluorescence tomographic slice of the partially crystallized 20/80 blend in Figure 9 illustrates clearly the tortuosity of SC lamellar growth caused by avoidance of regions with the pure enantiomer. Fluorescence images in Figure 10b, as well as the simultaneously recorded POM images in Figure 10a, show how the enantiomer then crystallizes within and between spherulites on further cooling.

Similar situations may occur in other binary systems when diffusion is slower than phase growth. A related effect has been shown recently in a liquid crystal racemate, forming a locally deracemized phase (space group *Fddd*). This phase consists of adjacent right- and left-handed helical columns containing separated pure chiral enantiomers. While the racemic high-temperature columnar phase forms from the melt within 20 ms, the local deracemization and formation of the *Fddd* phase take 20 s.^40^ In this case, the rate-determining step is unpairing, while in the PLA case, it is pairing. In the liquid crystal, the unpairing occurs in a viscous columnar liquid crystal phase, hence the slow diffusion, while in PLA, even in the melt, the diffusion is sufficiently slow to severely disrupt the pairing needed for unhindered SC growth.

Worth noting is also the sawtooth appearance of lamellar edges seen most clearly in the inset in Figure 11b and indicated also elsewhere in AFM images of blends as periodic protrusions in edge-on lamellae. The recesses between these teeth are caused by pinning due to accumulated “impurity”, i.e., the excess pure enantiomer. Deep but irregular recesses caused by impurity pinning, described as “fjords”, have also been observed in some lysozyme crystals.^41^ Fjord-like bays have also been seen in Monte Carlo simulations of extended-chain growth of long alkane crystals pinned by folded-chain “self-poison”.^42^ In the latter case, the depth of the “fjords” was due to the limitations of the unidirectional radial “solid-on-solid” growth model used. In SC growth of PLA, the growth also occurs tangentially, thus partially filling the “fjords” by side-way (tangential) growth. This resulted in relatively shallow bays and faceted serrated lamellar edges. The observed periodicity of the “saw teeth” is intriguing and calls for further investigation.

### 4.2 Composition Dependence of Birefringence

The strong dependence of birefringence on the L/D ratio is discussed next. The weaker birefringence of SC spherulites of the “ideal” 50/50 composition compared to that of HC spherulites of pure enantiomers has already been discussed above. As depicted schematically in Figure 11a, the further decrease in brightness on moving away from the 50/50 composition is due to three additional causes. First, in the range of L/D 30/70, the lamellae deviate further away from the radial direction (see Figure 11c and the FT inset), with evidence of some epitaxial lamellar branching. Second, in the 20/80 region, the POM brightness is reduced even more, affected by many lamellae turning flat-on, parallel to the substrate. At least some of these lamellae are thought to be HC grown from the now less abundant SC nuclei (Figure 11d). As the HC α-form is optically weakly biaxial and the projection of the slow axis on the lamellar plane is parallel to the radial growth direction,^43^ there is some cancellation of the tangential slow axis of edge-on radial lamellae, leading to virtual extinction in the 20/80 L/D range. Incidentally, crystallinity in this composition range is at its minimum; see Figure 8c,g. Moving on to the edge of the contact zone, to the region of >90% enantiomeric purity, the low birefringence here seems to be mainly due to the subwavelength size of the small axialites, primarily HC. Full brightness returns as the area of larger edge-on lamellae of spherulites of the pure enantiomer is reached.

An interesting anomaly is observed during slow cooling the contact sample from the melt as temperature reaches 120 °C; see Figure 3c. A narrow birefringent strip with spherulites appears amid a dark isotropic melt at around 3 and 97% PDLA. When cooling was continued, the strip merges with the bright spherulitic texture of pure enantiomers (Figure 3d). This can be seen in Figure 6a as a delayed ascent of the 99% curve. The isolated bright strip is were HC spherulites grew on a few SC nuclei that succeeded to form in pockets still sufficiently rich in the counter-enantiomer. While the SC nuclei could not grow further, HC spherulites could, as enantiomeric purity was sufficient for their growth but not for their nucleation. However, at 120 °C, in the region of still higher enantiomeric purity, the concentration of the counter-enantiomer was too low to allow SC nucleation, yet too high to allow HC growth, hence the dark band between the bright strip and the bulk HC area. It is unlikely that this anomaly would have been noted without the continuous composition gradient of the contact sample.

## 5 Conclusions

In conclusion, contact specimens of PLLA and PDLA were studied by POM, μ*-*WAXS, and AFM, complemented by POM, fluorescence microscopy, and DSC experiments on separate blends of selected compositions. The L/D ratio of the precursor melt was determined by detection of optical rotation. The continuous change in composition enabled reliable determination of changes in morphology resulting exclusively from changes in the enantiomeric ratio. Remarkable morphological differences were observed between pure enantiomers and the blends. In particular, blend morphology showed clear evidence of “poisoning by purity” of SC crystallization at all blend compositions. The gradation of birefringence from moderate at L/D 50/50 to zero around 90/10 could be attributed reliably to a succession of different causes: meandering radial edge-on lamellae, fully random meandering edge-on lamellae, flat-on lamellae, and submicron-sized axialites. A feature of the binary phase diagram is pure enantiomers acting as impurity to the SC and counter-enantiomer acting as impurity to homocrystallization of the enantiomers.

## 6 Experimental Section

### 6.1 Materials

PLLA and PDLA were purchased from Jinan Daigang Biomaterial Co., Ltd. and used without further purification. The weight-average molecular weight (*M̅*_w_) and polydispersity (PDI) of PLLA and PDLA were both 54 kDa and 1.7, respectively, according to gel permeation chromatography (see Figure S5 and calculation in the SI). Dichloromethane purchased from Sinopharm Chemical Reagents was used as received. Nile Red was purchased from Sigma–Aldrich.

### 6.2 Preparation of Uniform Films

Film samples of PLLA, PDLA, and PLLA/PDLA blends were prepared by solution casting. Films of pure enantiomers were prepared by dissolving the polymers in dichloromethane (0.01 g/mL) at room temperature under stirring. 10 mL of the solution was cast onto a Petri dish, which was then left to dry in air for 24 h. The films were finally dried under vacuum at 50 °C for 12 h. PLLA/PDLA blends with different PDLA contents (50, 60, 70, 80, 90, and 95%) were also prepared in a similar way, with PLLA and PDLA first dissolved in dichloromethane separately before solution blending. For the dye-doped sample, 0.05 wt % Nile Red was dissolved in the PLLA/PDLA mixed solution before casting.

### 6.3 Characterization

#### 6.3.1 Crossed- and Decrossed-Polarized Optical Microscopy

An optical microscope (Olympus BX51-P) was used, equipped with a Linkam LTS420E hot stage and T95-HS controller. A λ-plate (530 nm) was used to determine the direction of the slow axis. A Berek compensator was used to measure the birefringence of SC and HC spherulites. Decrossed POM was performed by rotating the analyzer away from 90° to polarizer. For details, see Section 2 and Figure 2.

#### 6.3.2 2D and 3D Fluorescence Microscopy

Fluorescence microscopy (FM) images were acquired on an Olympus BX51-P microscope in reflection mode utilizing a CoolLED pE-300 white light source, a BP 460–490 excitation filter, a DM 500 dichromatic mirror, and an LP 520 emission filter. For 3D imaging, an upright Zeiss LSM 510 META confocal microscope equipped with a Ti-sapphire multiphoton laser was employed to capture images at various depths within the sample. The excitation wavelength was set at 1000 nm, and the fluorescence emitted by NR was collected through a BP 565–615 bandpass filter.

#### 6.3.3 Microbeam WAXS

WAXS patterns were recorded at room temperature using a custom-designed microbeam WAXS system (Xenocs, France) at Changchun Institute of Applied Chemistry, Chinese Academy of Science. A Cu Kα beam from a microfocus tube was focused using a GeniX 3D multilayer mirror on the sample, where the beam cross-section was 40 × 60 μm^2^ (H × V). The distance from the sample to the detector (Pilatus 100 K, Dectris) was 68 mm. The sample was moved from pure PLLA to pure PDLA, across the X-ray beam in 40 μm steps. Exposure time at each point was 5 min. The % crystallinity of SC (*X*_SC_) and HC (*X*_HC_) was determined using  and , respectively, where *A*_SC_, *A*_HC_, and *A*_amorph_ are the sums of integrated intensities of all Lorentz-corrected diffraction peaks of the SC form, HC form, and the amorphous phase, respectively. An example of the curve resolution of a WAXS profile is shown in Figure S6 of the SI.

#### 6.3.4 Differential Scanning Calorimetry

A TA Instruments DSC250 calorimeter, calibrated with indium, was used to record crystallization and subsequent melting of PLLA, PDLA, and PLLA/PDLA blends with different discrete L/D ratios. The thermal program for crystallization was the same as the “cool (110)–isothermal” route in Figure 1. % crystallinity of SC or HC was calculated, as usual, as , where Δ*H*_m_ represents the melting enthalpy of the SC and HC. Δ*H*_m_^0^ represents melting enthalpies of fully crystalline SC and HC equal to 142^44^ and 93 J/g,^45^ respectively. Since melting peaks of SC and HC are not overlapped, for samples containing both SC and HC, the total crystallinity could be calculated as .

#### 6.3.5 Atomic Force Microscopy

Tapping mode AFM experiments on “cool (130)–quench–anneal” and “cool (110)–quench–anneal” samples were performed by using a JPK Bio AFM instrument at 25 °C. A VTESPA-300 visible apex probe with a tip radius of less than 8 nm was used to target the features, which could be observed directly under the optical microscope attached to the AFM. The cover glass was removed from the sample before scanning.

## Supporting Information Available

Decrossed POM of the molten PLLA + PDLA blend; WAXS profile of 110/200 reflection; AFM height images; and GPC details and correction (PDF)
