# Supplementary material for: Continuous Spectrum of Morphologies and Phase Behavior across the Contact Zone from Poly(l-lactide) to Poly(d-lactide): Stereocomplex, Homocrystal, and Between
Source: Macromolecules. 2023 Oct 27;56(21):8754–66. doi: 10.1021/acs.macromol.3c01815 (PMC10653275; doi:10.1021/acs.macromol.3c01815)
Supplement: Supplementary file 1 — ma3c01815_si_001.pdf [file ma3c01815_si_001.pdf]

Supporting Information

Continuous Spectrum of Morphologies and Phase Behavior across Contact Zone from Poly(L-lactide) to Poly(D-lactide): Stereocomplex, Homocrystal, and Between

Jiaming Cui[1], Shu-Gui Yang[1,], Ruibin Zhang[2], Yu Cao[1], Yubo Wang[1], Xiangbing, Zeng[2], Feng Liu[1,*], and Goran Ungar[1,2,*]*

[1] Shaanxi International Research Center for Soft Matter, State Key Laboratory for Mechanical Behavior of Materials, Xi'an Jiaotong University, Xi'an 710049, P.R. China
[2] Department of Materials Science and Engineering, Sheffield University, Sheffield S1 3JD, U.K.

1. Decrossed POM study of molten PLLA and PDLA

Brightness of molten PLLA and PDLA films was measured by decrossed POM, whereby the analyzer was rotated from -12° to +12° away from the crossed orientation in 1° steps while images were taken in rapid succession, as shown in **Figure S1a**. **Figure S1b** shows the difference in brightness between PLLA and PDLA, reaching its maximum and minimum value at -4° and +4°, respectively.

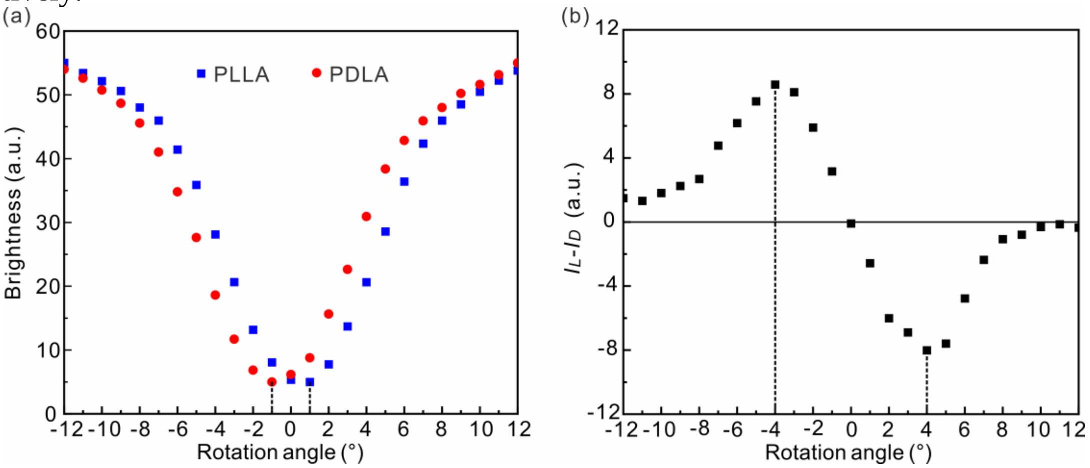

Figure S1. (a) Brightness of polarized micrographs of molten PLLA and PDLA under different analyzer rotation angle away from the crossed orientation. (b) Difference in brightness between PLLA (I_L) and PDLA (I_D) under different rotation angles of the analyzer.

2. 1D microbeam WAXS profile of "cool(110)-isothermal" sample

Figure S2 shows the characteristic reflections of $HC_{(110)/(200)}$ of "cool(110)-isothermal" sample. The reflections at 2θ = 16.75° and 110 °C indicate the coexistence of the α' and α form HC.[S1,S2]

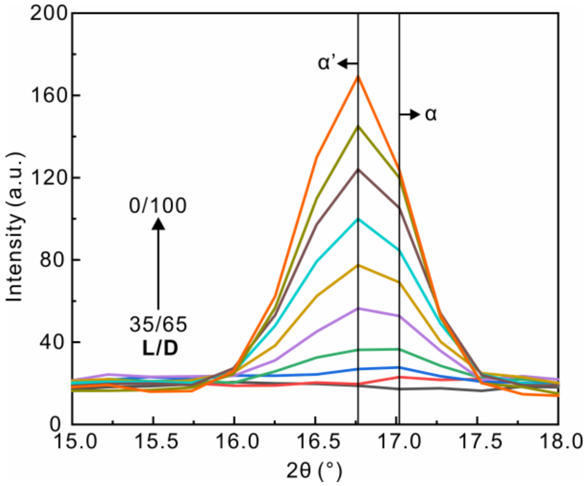

Figure S2. 1D WAXS profile recorded at different positions on the "cool(110)-isothermal" sample, showing the overlapping (110)/(200) reflections of the α and α' (or δ) forms.

3. AFM height images of “cool(130)-quench-anneal” sample

Figure S3 shows AFM height images of “cool(130)-quench-anneal” sample recorded at different L/D ratio along the composition gradient.

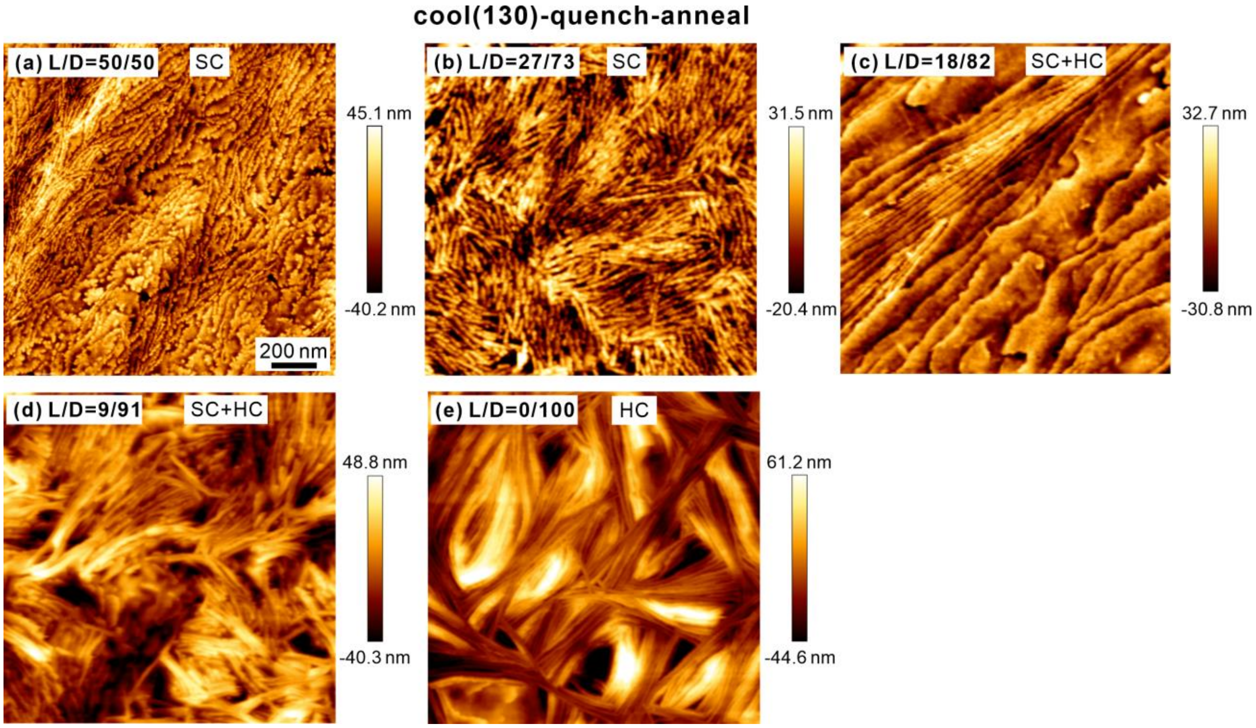

Figure S3. AFM height images of “cool(130)-quench-anneal” sample recorded at positions with different L/D ratios. (a) 50/50, (b) 27/73, (c) 18/82, (d) 9/91 and (e) pure PDLA.

4. AFM height images of “cool(110)-quench-anneal” sample

Figure S4 shows AFM height images of “cool(110)-quench-anneal” sample. The images were collected at positions in pure enantiomers and neighboring areas with a small amount of enantiomer impurity.

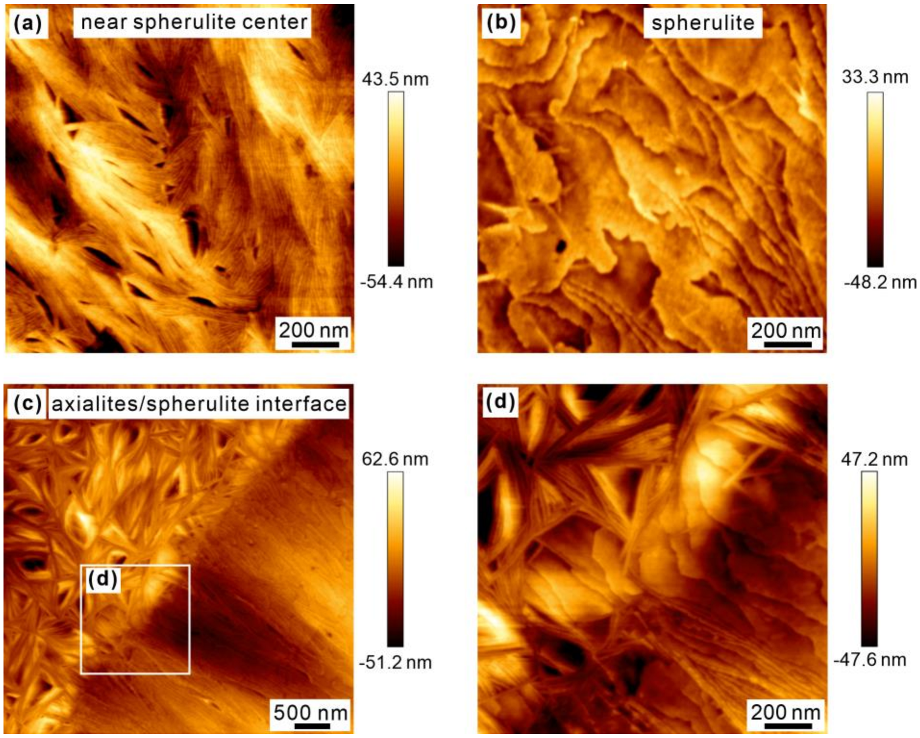

Figure S4. AFM height images of "cool(110)-quench-anneal" sample collected at different positions. (a) Near the center of spherulite; (b) dark area in the dark slice in POM in the northwest sector; (c) interface between spherulite and axialites. (d) Enlarged white rectangle in (c).

5. GPC of PLLA and PDLA

Molecular weight distribution of PLLA and PDLA was measured by GPC at 50 °C. Chloroform was used as the mobile phase. The GPC curves are shown in **Figure S5**.

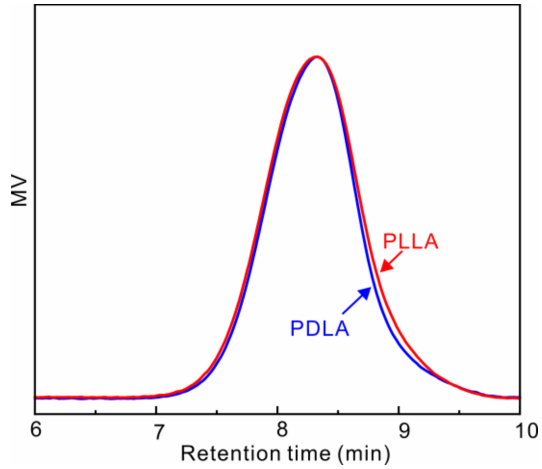

Figure S5. Gel permeation chromatograms of PLLA and PDLA.

Using narrow fractions of PMMA as standards, the weight-average molecular weight ($\overline{M}_w$) and polydispersity (PDI) of PLLA and PDLA were both obtained as 82 kDa and 1.7, respectively. However, to obtain a more accurate value of $\overline{M}_w$, a correction was applied for the difference in hydrodynamic volumes of PMMA and PLA, as only polymers with the same hydrodynamic volume will elute at the same retention time. Hence the molecular weight of PLA was corrected using the Mark-Houwink equation[S3]:

$$[\eta]_1 M_1 = [\eta]_2 M_2 \qquad (1)$$

and

$$[\eta] = KM^{\alpha} \qquad (2)$$

Then

$$K_1 M_1^{\alpha_1+1} = K_2 M_2^{\alpha_2+1} \qquad (3)$$

According to literature[S3], K and α of PMMA in chloroform at 53 °C are 3.9×10^{-3} mL/g and 0.82, respectively. For PLA in chloroform, we could only find the parameters obtained at 30 °C, where $K = 1.31\times10^{-2}$ mL/g and $\alpha = 0.78$.[S4] According to equation (3), the corrected $\overline{M}_w$ of our PLA polymers is about 5.4×10^{4} g/mol.

6. Curve resolution of WAXS profile

To evaluate the crystallinity of SC and HC at different position in contact samples, the microbeam WAXS profiles were resolved into individual Bragg components and the amorphous scattering curve using Origin. WAXS profile recorded in "cool(110)-isothermal" sample is shown as an example in **Figure S6**.

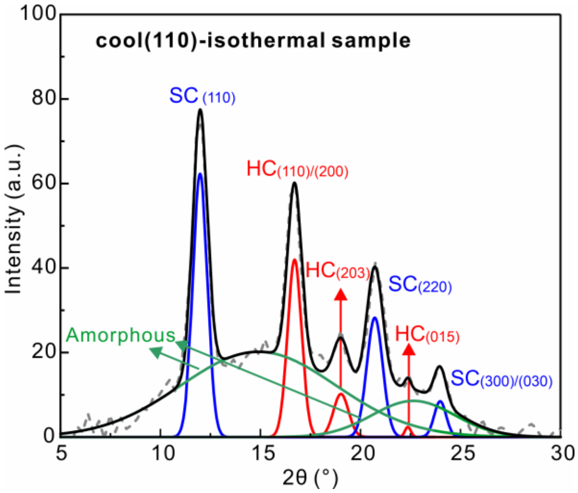

Figure S6. Peak-fitting of WAXS profile collected in "cool(110)-isothermal" sample.

References

(S1) Pan, P.; Zhu, B.; Kai, W.; Dong, T.; Inoue, Y. Effect of Crystallization Temperature on Crystal Modifications and Crystallization Kinetics of Poly(L-lactide). *J. Appl. Polym. Sci.* **2008,** *107*, 54-62.

(S2) Zhang, J.; Tashiro, K.; Tsuji, H.; Domb, A. J. Disorder-to-Order Phase Transition and Multiple Melting Behavior of Poly(l-lactide) Investigated by Simultaneous Measurements of WAXD and DSC. *Macromolecules* **2008,** *41*, 1352-1357.

(S3) Wagner, H. L., The Mark–Houwink–Sakurada Relation for Poly(methyl methacrylate). *J. Phys. Chem. Ref. Data* **1987**, *16*, 165-173.

(S4) Dorgan, J. R.; Janzen, J.; Knauss, D. M.; Hait, S. B.; Limoges, B. R.; Hutchinson, M. H., Fundamental Solution and Single-chain Properties of Polylactides. *J. Polym. Sci. B: Polym. Phys.* **2005**, *43*, 3100-3111.
